# Amplified MS2 labeling reveals heterogeneous dynamics of RNA granules in the live murine brain ex vivo

**DOI:** 10.1371/journal.pone.0361966

**Published:** 2026-11-05

**Authors:** Hyungsik Lim, Grace P. Cooper

**Affiliations:** School of Optometry, Indiana University, Bloomington, Indianapolis, United States of America; National Institutes of Health, UNITED STATES OF AMERICA

## Abstract

Dynamics of messenger ribonucleic acids (mRNAs) and the complexes with associated proteins (RNPs), also known as RNA granules, provide post-transcriptional control of gene expression. This regulation is crucial for cell-type diversity and activity-dependent plasticity of the mammalian central nervous system (CNS). However, elucidating the physiological significance of RNA granules has been hampered by technical difficulties to probe them in live brain. Here, we describe a novel method to visualize RNA granules in native CNS tissues of mouse. To amplify the fluorescence signal from single mRNAs incorporating MS2 stem loops, MS2 capsid protein (MCP) was conjugated with 4 tandem superfolder green fluorescent proteins (sfGFPs). Using MCP-4 × sfGFP, significantly more RNPs could be detected in the fresh acute brain slices than with MCP-GFP. Amplified MS2 signal could visualize *Actb* mRNAs in specific cells, either driven by promoter or via Cre-dependent expression, revealing remarkably distinct localization and movements of RNPs across different CNS cell types, including neurons and glial cells. The highly sensitive live-tissue RNA imaging could be useful for investigating the regulation of the dynamics of RNA granules in the native niche, ultimately in naive and diseased animal models.

## Introduction

Dynamics of ribonucleic acids (RNAs) are an element of post-transcriptional control of gene expression [1–4]. After the synthesis and processing of messenger RNAs (mRNAs), various types of ribonucleoprotein (RNP) complexes are assembled in the cytoplasm, also known as RNA granules, which modulate stability, trafficking, and translation of mRNAs [5–7]. Many aspects of RNA granules are still unknown, particularly the physiological significance in vivo, although their dysregulation is implicated in neurodegenerative disorders [8–10]. Transient RNA granules have been hypothesized to enable many polarized cells of the central nervous system (CNS), which is characterized by diversity of cell types and activity-dependent plasticity. To understand how dynamics of RNPs support these CNS characteristics and how failure could give rise to diseases, the molecules must be observed in their native environment where cellular identity and intrinsic physiology are preserved. The MS2 method allows nascent transcripts and endogenous mRNAs to be visualized in vivo by inserting multiple copies of stem loop of MS2 bacteriophage, i.e., MS2 binding sequence (MBS), into the gene of interest and expressing MS2 capsid protein (MCP) which targets the MBS, fused with green fluorescent protein (GFP) [11]. Then, the RNA of interest binds numerous MCP-GFPs, amplifying fluorescent signals for detection. Recently, knock-in mouse strains have been created employing the MS2 strategy for elucidating transcriptional dynamics of various genes [12,13].

Despite its widespread success in live-cell studies, however, the conventional MS2 method is not well-suited for visualizing RNP particles in thick brains. Due to turbidity degrading the resolution and signal-to-background ratio, the sensitivity is often not sufficient for imaging single RNP particles in fresh ex vivo tissue or live animals. As a result, the number of RNP particles seen by live-tissue MS2 imaging has been consistently much smaller than anticipated from in vitro measurements, such as fluorescence in situ hybridization (FISH). An unrepresentative subpopulation of RNP particles due to undersampling could introduce significant biases, hampering accurate characterization of the in vivo function of RNA granules.

Here we present a novel MS2 labeling technique that increases sensitivity for detecting endogenous mRNAs in fresh murine tissues. The fluorescence from individual mRNAs is amplified by tagging MCP with 4 tandem GFPs, sufficient for visualizing single RNP particles in the turbid tissue. Enhanced sensitivity is demonstrated for measuring the dynamics of *Actb* mRNAs in neuronal and glial cells, validating an in vivo RNA labeling suitable for unraveling RNA granules in the context of pathophysiology of the mammalian brain.

## Results

### Sensitive detection of RNA granules in fresh brain slices

To increase fluorescence per individual RNA molecule, we tagged MCP with four copies of GFP (Fig 1(a)). These tandem GFPs provide compounded amplification orthogonal to the number of MBS repeats. To minimize the risk of forming RNA aggregates, superfolder GFP (sfGFP) was used, i.e., a GFP variant with robust folding mechanics [14]. For delivering MCP-4 × sfGFP into live tissues, an adeno-associated virus (AAV) vector was designed with the human ubiquitin C (UbC) promoter and the woodchuck hepatitis posttranscriptional regulatory element (WPRE). The number of tandem GFPs was limited by the capacity of AAV packaging. The MCP-4 × sfGFP sequence was approximately 3.3 kb and the total size of insert, including the inverted terminal repeats (ITRs), was ~ 4.9 kb. To target *Actb* mRNAs, a knock-in mouse strain *Actb*^*MBS/MBS*^ was employed, containing 24 repeats of MBS in the 3’ untranslated regions (UTR) of both *Actb* alleles [12]. As a result, each *Actb* mRNA could harbor up to 48 MCP-4 × sfGFPs, or a total of 192 sfGFPs. After stereotaxic injection of high-titer AAVs, the acute brain slices of the *Actb*^*MBS/MBS*^ mouse were imaged by two-photon microscopy (TPM). In the hippocampi expressing MCP-4 × sfGFP, bright fluorescent spots could be identified without additional image postprocessing (Fig 1(b)). By contrast, the *Actb*^*MBS/MBS*^ brain expressing MCP-1 × GFP displayed far fewer punctate features above the background. To confirm the specificity of fluorescent puncta, the images were compared to negative control, i.e., wild-type (WT) mice injected with MCP-4 × sfGFP. Bright spots were absent in the WT brain (Fig 1(c)), suggesting that the signals were associated with MBS, thus likely from *Actb* mRNAs. The intensity of particles was heterogenous, possibly due to the variable number of mRNAs. Their sizes were close to the optical resolution of microscopy, but the size alone could not determine whether some particles contained multiple mRNAs. *Actb* RNA granules exhibited an uneven spatial distribution in the hippocampus, being much more enriched in the somata than the neuropils. Most RNA granules were extremely mobile, but a small number of them were immobilized near the perinuclear space (Fig 1(d), S1 Movie). The movement of these RNA granules was confined primarily to the soma and also excluded from the intracellular organelles (Fig 1(d)). Notably, nuclear mRNAs were scarce and RNA granules in the dendrites were also relatively fewer than in the soma. The dendritic RNPs were much dimmer than somatic particles (Fig 1(e)), suggesting fewer copies of *Actb* mRNAs.

**Fig 1:**
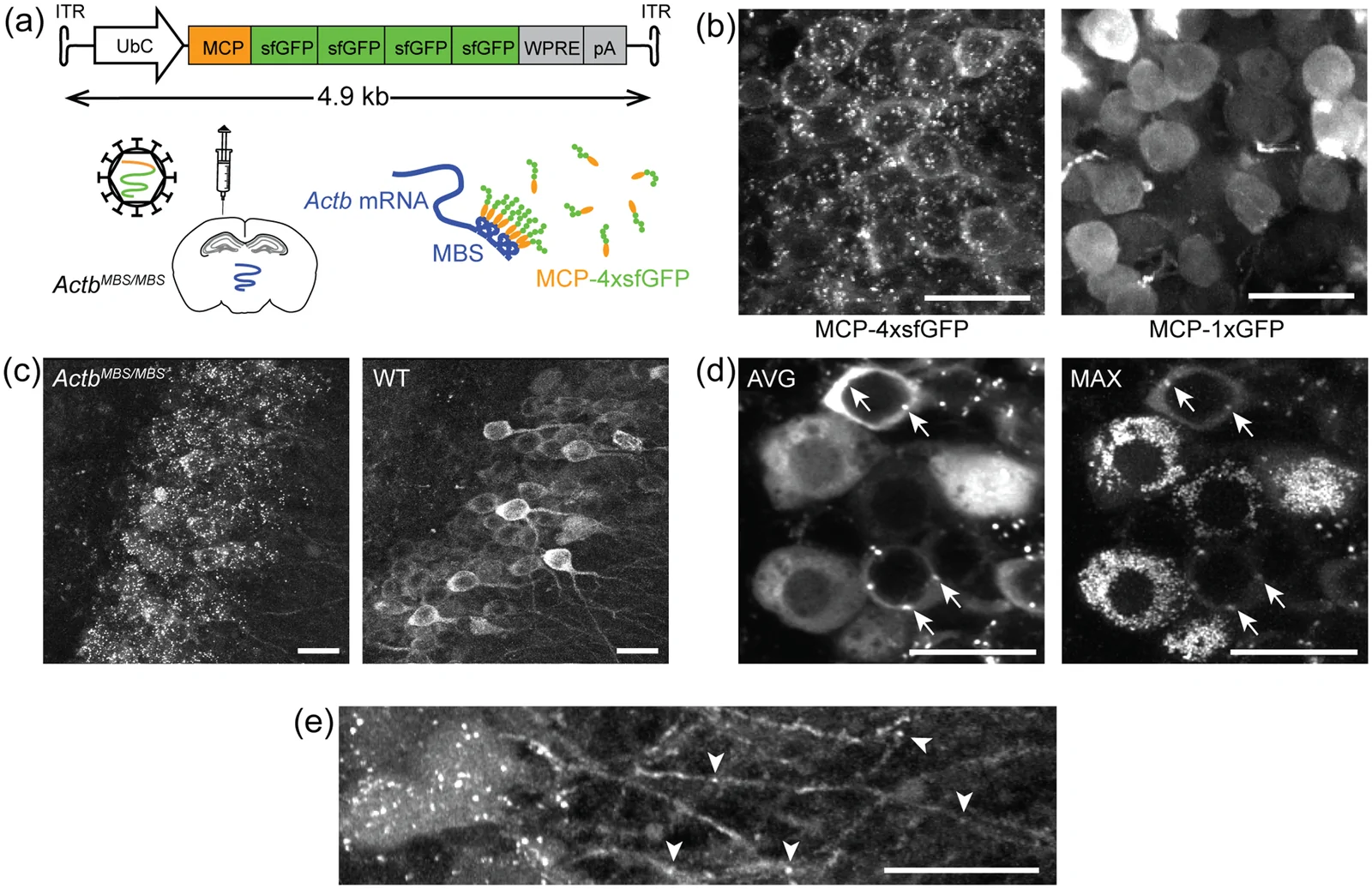
Amplified detection of mRNAs with MCP-4 × sfGFP. (a) The DNA construct and labeling procedure. pA, poly-adenylation signal. (b) The hippocampus of *Actb*^*MBS/MBS*^ mice expressing MCP-4 × sfGFP vs. MCP-1 × GFP. (c) *Actb*^*MBS/MBS*^ vs. WT mice expressing MCP-4 × sfGFP. (d) Average and maximum projections of time-lapse images of *Actb*^*MBS/MBS*^ brain expressing MCP-4 × sfGFP. Arrows, RNPs anchored near the perinuclear space. (e) RNPs in the dendrites (arrowheads). Scale bars, 20 µm.

### Cre-dependent MCP-4 × sfGFP for imaging RNP particles in specific cells

Dynamics of mRNAs are highly variable depending on the cell type, which is difficult to resolve with the UbC promoter driving the expression in multiple cell types. To isolate cell-type-specific dynamics of *Actb* RNPs, we engineered a Cre-dependent MCP-4 × sfGFP by flanking it in a double-floxed inverted open (DIO) reading frame [15], i.e., DIO-MCP-4 × sfGFP (Fig 2(a)). Consequently, while every *Actb* mRNA in the *Actb*^*MBS/MBS*^ mouse contains MBS, only those in *Cre+* cells were fluorescently labeled where DIO-NLS-MCP-4 × sfGFP was inverted by Cre-lox recombination. Also, a nuclear localization signal (NLS) was included to improve the contrast for cytoplasmic RNA granules by sequestering free MCPs to the nucleus [11]. A truncated WPRE, i.e., W3 [16], was employed to keep the total size of transgene below the AAV packaging capacity. The properties of neuronal RNA granules have been extensively studied [17–19]. To target them, *Actb*^*MBS/MBS*^ mice were crossed with *CaMKIIα-Cre* and *Grik4-Cre* strains [20,21]. Furthermore, for the visualization of RNA granules in glial cells, mice were used expressing Cre under the control of glial fibrillary acidic protein (Gfap), i.e., *Gfap-Cre* [22,23]. The hybrids that were homozygous in MBS and positive in Cre, referred to as *Actb*^*MBS/MBS*^*;CaMKIIα-Cre, etc* (Fig 2(b))*,* received AAV injection*.* Fig 2(c) depicts *Actb* RNPs in the *Cre+* cells within the CA3 regions. RNPs exhibited significant enrichments in the neuronal somata (Fig 2(c) and (d)), like Cre-independent MCP-4 × sfGFP. In *Actb*^*MBS/MBS*^*;Gfap-Cre* mice, however, it was difficult to delineate RNA granules specific to glial cells due to substantial Cre expression in nearby pyramidal neurons (Fig 2(e)).

**Fig 2:**
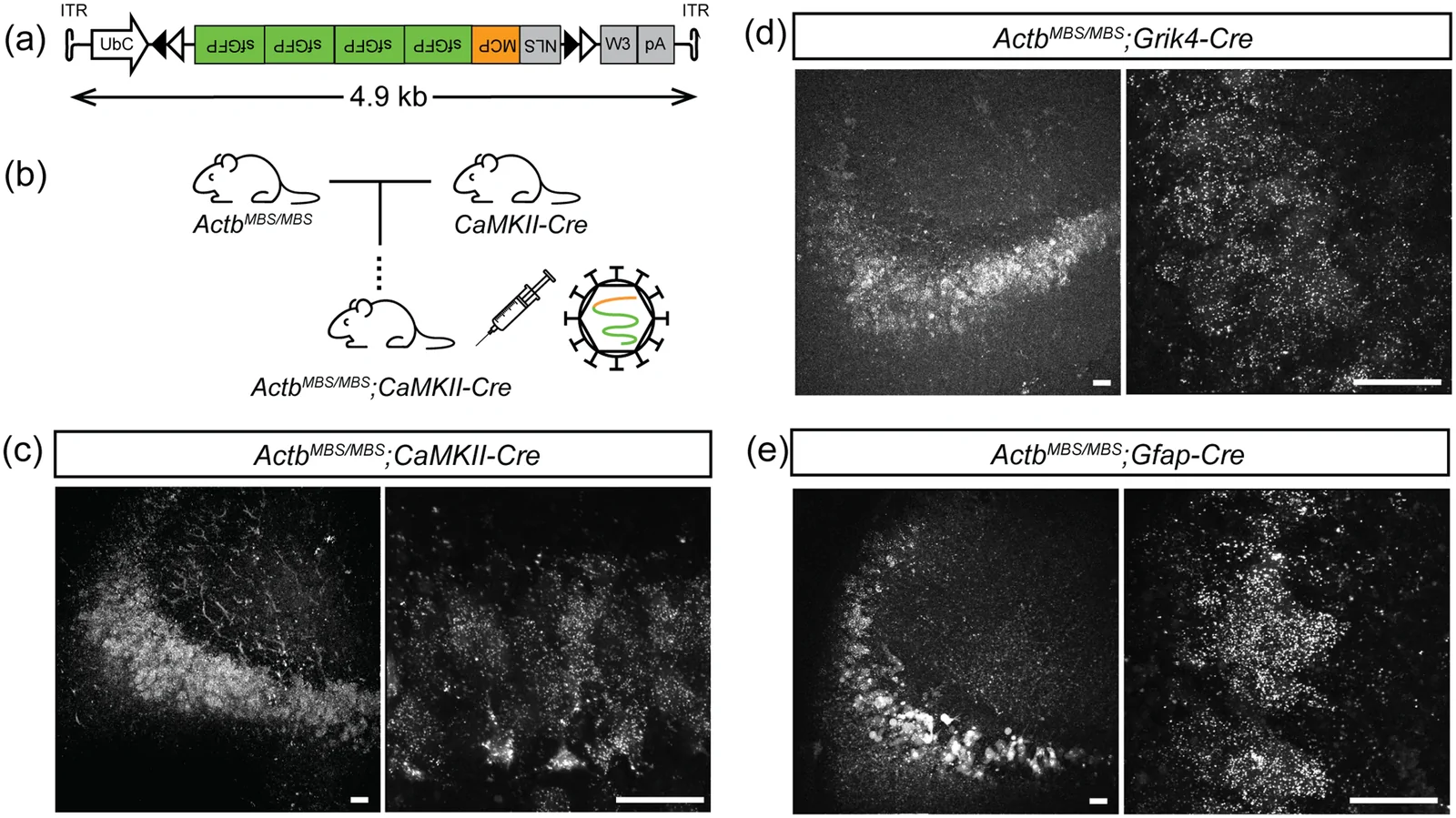
Visualizing *Actb* mRNAs in specific cells. (a) The DNA construct for Cre-dependent MCP-4 × sfGFP. (b) The creation of hybrid *Actb*^*MBS/MBS*^ Cre mouse strains. (c-e) *Actb* mRNAs in the *CaMKIIα-Cre* + , *Grik4-Cre* + , and *Gfap-Cre+* cells of the hippocampus. Scale bars, 40 µm.

### Highly sensitive visualization unveiled heterogeneous composition of neuronal *Actb* RNA granules in the hippocampus

With the ability to visualize *Actb* mRNAs in specific cells, their properties could be measured free from the variability across cell types. We compared the number of neuronal RNA granules that can be detected by UbC-DIO-NLS-MCP-4 × sfGFP versus Ef1α-DIO-MCP-1 × GFP (without NLS). AAVs carrying each construct were injected*,* and acute brain slices were imaged by TPM. Representative images of the hippocampi of *Actb*^*MBS/MBS*^*;CaMKIIα-Cre* are shown in Fig 3(a) (max projections of 12-µm z-stacks). For precise quantification of the image contrasts, RNA particles and the soma background were segmented (Fig 3(b)). RNA particles were detected by peak finding. Due to low contrasts of the spots visualized by MCP-1 × GFP, the confidence for positive identification as RNA granules was poor, thus likely inaccurate, but they were far less ambiguous by NLS-MCP-4 × sfGFP. The number of detected puncta was nearly thirteen times higher with NLS-MCP-4 × sfGFP compared to MCP-1 × GFP, although the latter might not be all RNA granules (Fig 3(c)). The improved performance may not be solely due to the copy number of GFP but there could be also other contributing factors, as they were different in their promoters, the presence/absence of NLS, AAV serotypes, and titers. The intensity of RNA particles labeled by NLS-MCP-4 × sfGFP, relative to the somatic background, was on average 2.75 times higher than those labeled by MCP-1 × GFP. The factor of amplification was lower than 4 possibly due to the different backgrounds from free MCP reporters. Plausibly, it might also arise from the composition of the visualized RNA particles. The intensity histogram showed that RNA particles labeled with NLS-MCP-4 × sfGFP had multiple distinct subpopulations (Fig 3(d)), indicating neuronal RNA granules were heterogenous. In comparison, the histogram of RNA particles labeled by MCP-1 × GFP did not show discernible subpopulations, likely because only a subset of RNPs containing many *Actb* mRNA molecules were visualized. Omitting small RNPs by MCP-1 × GFP could reduce the apparent gain of NLS-MCP-4 × sfGFP. To analyze the subpopulations of RNA particles quantitatively, the intensity distribution of NLS-MCP-4 × sfGFP puncta was fitted to a Gaussian mixture model. Each Gaussian has mean, covariance, and weight as free variables, and the number of Gaussians was determined by the maximum likelihood, i.e., one that minimizes the Akaike information criteria (AIC) value. The five most significant components (arrowheads, Fig 3(d), out of 9 components) displayed multiple intensity subpopulations with approximately equal mode spacing. Gaussian mixture model has been applied for quantitative analysis of single-molecule FISH (smFISH), where mRNA copy numbers are determined with an assumption that cytoplasmic signals represent the intensity of single transcript [24,25]. In the images of NLS-MCP-4 × sfGFP, however, the cytoplasmic background (unity in Figs 3(c) and (d), magenta) was comparable to the mode spacing. As a result, the image-based reference of single copy was not afforded to verify that the uniform increment of intensities was indeed due to the integer multiples of *Actb* mRNAs inside the RNPs.

**Fig 3:**
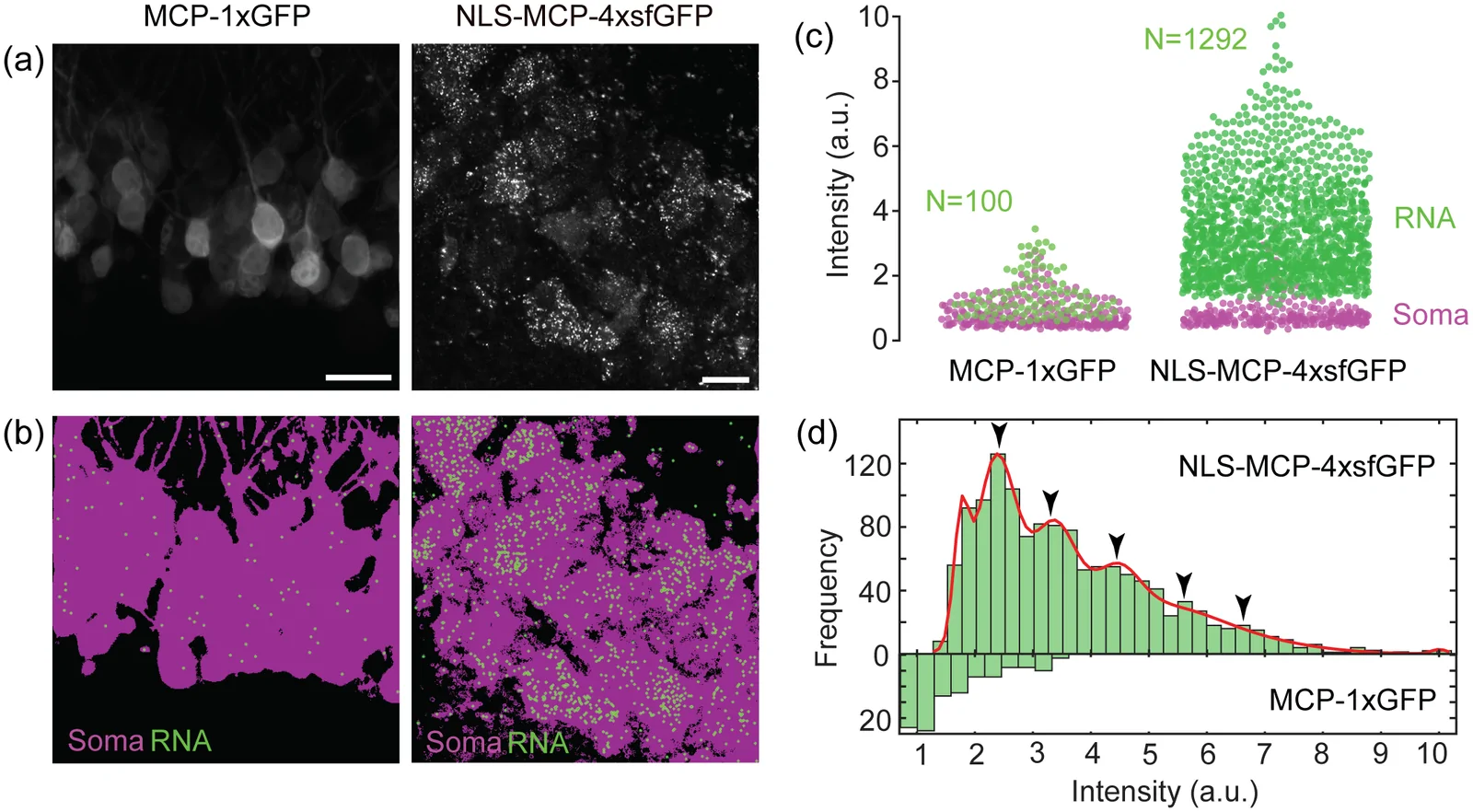
Enhanced sensitivity for quantifying *Actb* mRNAs in neuronal RNA granules. (a) *Actb*^*MBS/MBS*^*;CaMKIIα-Cre* brains expressing MCP-1 × GFP vs. NLS-MCP-4 × sfGFP, differing in their promoters, AAV serotypes, and titers, as described in the text. Max projections of 12-µm z-stacks. Scale bars, 20 µm. (b) Segmentation of RNA particles (green) and somatic background (magenta). (c) The intensity distribution of RNA particles (green) vs. somatic background (magenta). (d) Histogram of the normalized intensity of detected RNA particles. Red line, the maximum likelihood Gaussian mixture model. Arrowheads, 5 most significant modes at 2.34, 3.36, 4.47, 5.21, and 6.42.

### Movements of neuronal RNA granules were mostly stochastic

Next, movement of neuronal RNA granules was analyzed to compare with the prior findings from live-cell studies. In hippocampal *CaMKIIα+* and *Grik4* + neurons, *Actb* mRNAs were largely somatic and exhibited random motion (Fig 4(a), S2 Movie). The entire cytoplasm minus organelles was accessible to mobile RNA granules (Fig 4(b)). We asked whether the diffusive movement of *Actb* RNA granules depended on the cytoskeleton, e.g., dynamic actin filaments or the integrity of microtubules, or motor proteins. To address this, pharmacological agents were employed, including Jasplakinolide, an actin stabilizer, and nocodazole, a microtubule depolymerizer. Tetracaine was also used as a promiscuous inhibitor of kinesin motor proteins [26]. We anticipated that the effects of drugs, if any, would be to halt the motions immediately and evidently. However, under the condition employed in our time-lapse experiments, we did not find qualitative changes in the random motions of *Actb* mRNAs in response to the 1-hour treatment with 10-µM Jasplakinolide or 33-µM nocodazole (Fig 4(c)). Similarly, intervention with 5-mM tetracaine for >1 hour did not visibly alter the gross dynamics. The diffusive movements of *Actb* RNPs were analyzed quantitatively by single-particle tracking (SPT) (Fig 4(d)). Due to short track lengths arising from the 2D image plane sampling the 3D movement of RNP particles, it was more appropriate to evaluate the jump distance distribution, rather than the mean square displacement, which can resolve distinct mobile populations undergoing normal diffusion [27,28]. The best fit to a 2D diffusion model yielded two distinct species with diffusion constants of 0.026 and 0.68 µm^2^/sec with fractions of 21 and 79%, respectively (Fig 4(e)). The diffusion constant of mobile species was consistent with the range determined previously using live *Actb*^*MBS/MBS*^ cells [29], supporting that the diffusive movement of *Actb* RNPs was not altered significantly by binding with multiple copies of MCP-4xsfGFP.

**Fig 4:**
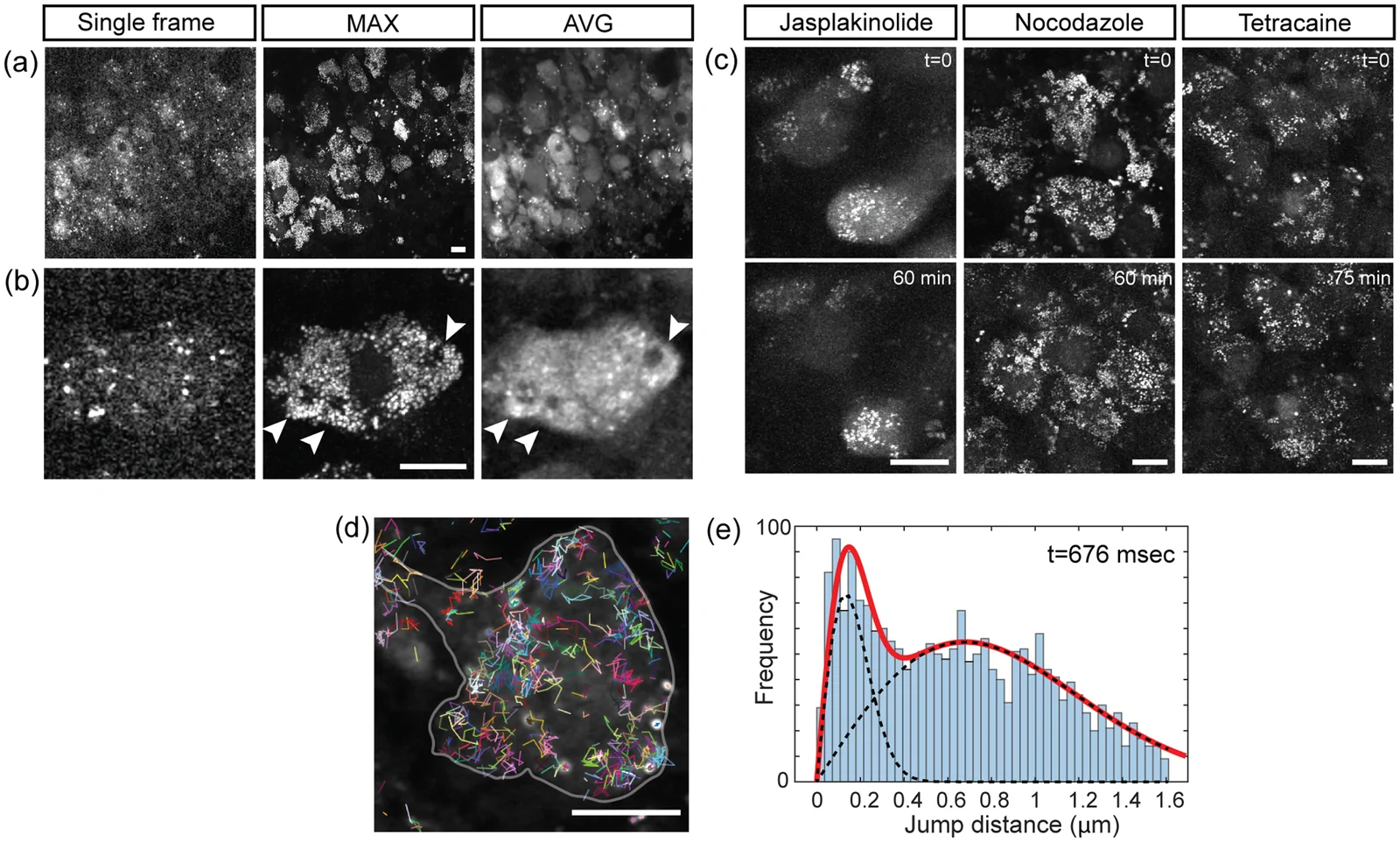
Movements of neuronal RNA granules. (a), (b) Single frame, maximum and average projections of time-lapse images of MCP-4 × sfGFP expressing cells in the *Actb*^*MBS/MBS*^*;Grik4-Cre* hippocampus (time = 162 sec). Arrowheads, cytoplasmic compartments limiting the access of RNPs. (c) Max projections of representative time-lapse images before and after the treatments of Jasplakinolide (10 µM), nocodazole (33 µM), and tetracaine (5 mM). (d) Single particle tracking traces overlaid with average projection. (time = 33.8 sec). (e) Jump distance distribution with the best fit to two-component 2D diffusion model (red). Dashed lines, each component. Scale bars, 10 µm.

### Glial *Actb* RNA granules exhibited distinguishable dynamics

Localization and local translation of *Actb* mRNAs have been studied extensively in cells whose in vitro culture systems are well established, e.g., neurons and fibroblasts [30,31]. However, much less is known about their dynamics in most other cell types. Evidence suggests mRNAs of various genes also localize in other polarized cells of the brain, e.g., radial glia [32], oligodendrocytes [33], and astrocytes [34,35]. On account of questions regarding the in vitro cultures recapitulating the actual physiology of the CNS, it is desirable to interrogate these non-neuronal mRNA dynamics in the native CNS tissue. Equipped with the improved sensitivity of MCP-4 × sfGFP, we decided to examine whether *Actb* mRNAs are localized in glial cells of the live brain tissue. As an alternative to *Actb*^*MBS/MBS*^*;Gfap-Cre*, which expressed Cre in neurons as well (Fig 2(e)), we employed a synthetic Gfap promoter, i.e., GfaABC1D [36]. The AAV for delivering GfaABC1D-MCP-4 × sfGFP (without an NLS) was produced (Fig 5(a)). After AAV injection, the acute brain slices were imaged. RNA granules in *Gfap+* cells of the hippocampus were visualized as punctate dots and their identity as *Actb* RNPs was confirmed by negative control with WT (Fig 5(b)). Interestingly, *Actb* RNPs exhibited dynamic properties that were visibly distinguishable from those in neurons. Whereas neuronal RNPs were mostly enriched in the cytoplasm, in glial cells a substantial fraction of them were localized to the distal processes. Also, regardless of localization, i.e., in the soma or processes, *Actb* RNA granules were mostly stationary exhibiting diffusive motions in few *Gfap+* cells (S3 Movie). *Gfap+* cells are inhomogeneous, encompassing multiple cell types across the brain. To dissect mRNA dynamics in a region-specific manner, two characteristic *Gfap+* cells were examined in the native niche, e.g., mature astrocytes in the CA1 and radial glia-like cells in the subgranular zone (SGZ) of the dentate gyrus (DG). The latter is adult neural stem cells so we anticipated the dynamics of RNPs therein might emulate that of developing neurons [30] rather than terminally differentiated neurons. Both astrocytes and radial glia-like cells exhibited substantial localization of *Actb* RNPs (Fig 5(c)–(f)). In the radial glia-like cells, modulated intensities within the processes suggested relatively small copy numbers of *Actb* mRNAs (Fig 5(e) and (f)). It proves that MCP-4 × sfGFP expands the library of cells in which mRNA dynamics can be documented.

**Fig 5:**
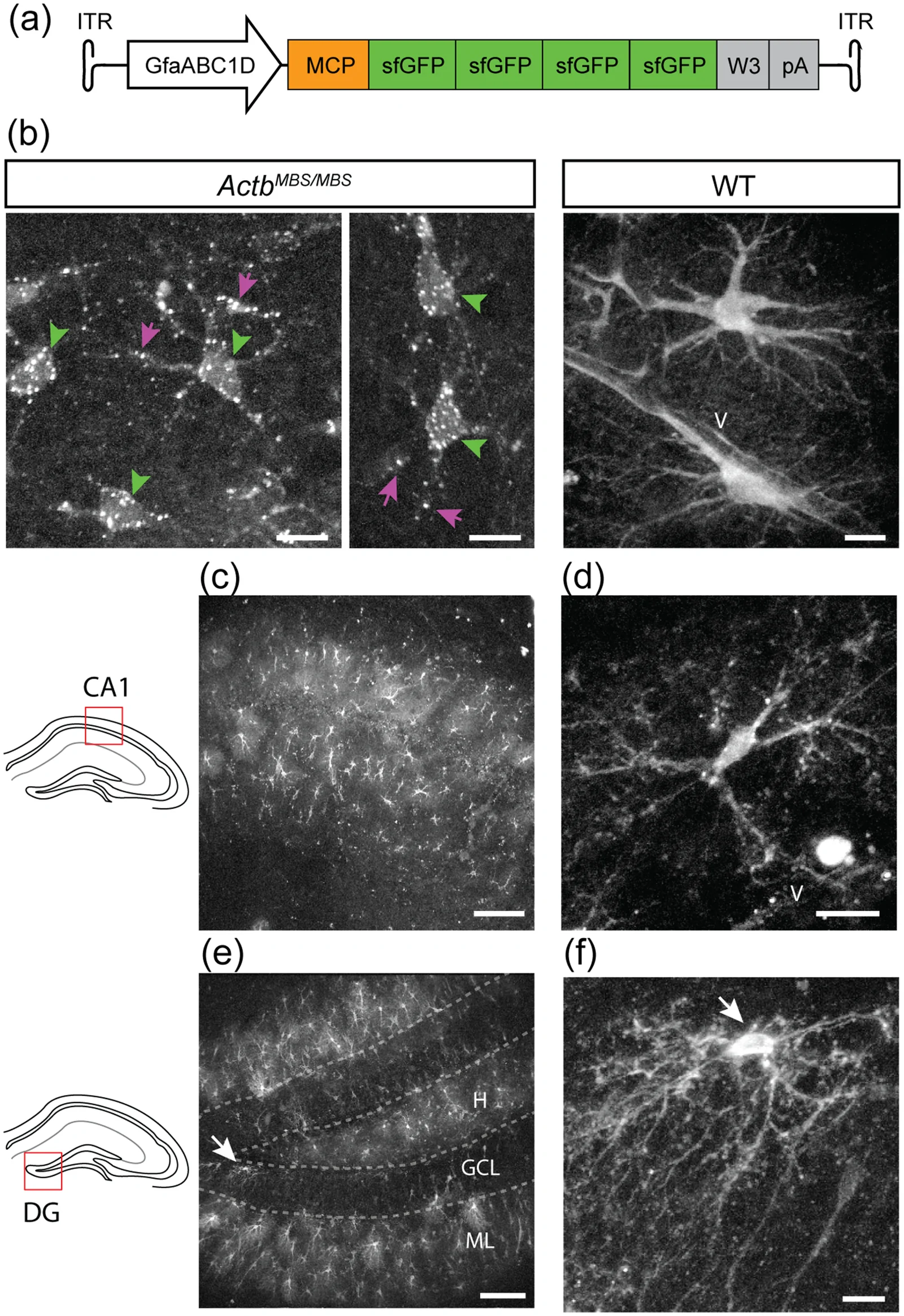
Visualizing *Actb* RNPs in the hippocampal glial cells. (a) The DNA construct for expressing MCP-4 × sfGFP under the control of GfaABC1D promoter. (b) *Actb*^*MBS/MBS*^ vs. WT mice expressing GfaABC1D-MCP-4 × sfGFP. *Actb* RNPs in the soma and processes (arrowheads and arrows, respectively). V, blood vessel. (c) MCP-4 × sfGFP in *Gfap*+ cells of the CA1 region. (d) Mature astrocyte in CA1. (e) MCP-4 × sfGFP in *Gfap*+ cells of the DG region. H, hilus; GCL, granular cell layer; ML, molecular layer. (f) Radial glia-like cell in the SGZ (arrow). All intensities are shown on the log scales. Scale bars: 100 µm ((c), (e)) and 10 µm (all others).

## Discussion

We have presented a new MS2 labeling strategy for upgrading fluorescence from single RNA molecules. With new MCP reporters containing 4 copies of sfGFP for enhancing MCP signals, as well as appropriate promoters, NLS, AAV serotypes, and titers, a larger group of RNPs could be detected in the murine brain than previously possible, which is a significant advance over live-tissue RNA imaging based on MCP-GFP [37,38] and comparable to in vitro RNA labeling, such as FISH, for revealing a comprehensive population of RNPs. Still, there could be potential biases in the visualized RNPs against the ground truth of the population. Notably, a subset of RNA granules with one or two mRNAs, an important class of functional RNA particles, e.g., in neurites [39], could not be detected efficiently. To capture the full RNPs, MCP signals could be increased further with more GFPs in tandem, which is feasible with lentiviral vectors providing a larger packaging capacity (8−12 kb). However, there are also caveats: A reporter protein larger than MCP-4 × sfGFP (~125 kDa) may be difficult to pass through the nuclear pore [40], compromising the efficiency of targeting nuclear RNPs. Furthermore, binding with numerous bulky MCP proteins could hinder or alter the movement of mRNAs. Lastly, unusually large reporter proteins could increase the potential for non-physiological artifacts, such as RNA aggregates [41].

Our method is analogous to another recent technique of amplifying MS2, in which each mRNA can be labeled with up to 384 GFPs for 8 MBS repeats [42]. Utilizing the Suntag system [43], the method conjugates multiple GFPs per MCP protein via two separate modules, i.e., MCP-24 × Suntag and single-chain variable fragment antibody tagged with GFP (scFv-GFP). As a result, attaining the predicted contrast hinges on balancing the bipartite stoichiometry, i.e., 24 scFv-GFP per MCP. MCP-4 × sfGFP does not have such requirements.

With MCP-4xsfGFP and live-tissue imaging, we have acquired data consistent with the previous studies employing FISH or live-cell imaging of primary or cultured cells, e.g., somatic neuronal RNA granules display random diffusive motions, which do not appear to be affected significantly by disruptions in the cytoskeleton or motor proteins. However, there were also results diverging from the previously documented behaviors [44–47]. For instance, while live-cell imaging has found that directional motion and localization of *Actb* mRNAs are mediated by active transport [47,48], we have found *Actb* RNPs are relatively scarce in the neuropils of the hippocampus and there are few motor-driven transports or directional *Actb* mRNAs. Our frame rate, approximately 1 Hz of typical scanning beam microscopy, was much slower than the previous studies employing wide-field illumination so the temporal resolution may not be adequate to capture brief transport on shorter time scales. Unlike in live-cell imaging where the full neurite lies within a field of view, the spatial range was limited for tracing directional RNA granules due to the 2D image plane intersecting the 3D tissue. It is also conceivable that directional motions were exhibited primarily by RNPs containing one or two copies of mRNAs, which did not register with MCP-4 × sfGFP. In addition to these technical reasons, it could be also due to the low frequency of long-range active transport. In a steady state of differentiated neurons in vivo, a nonnegligible fraction of directional RNPs would lead to an imbalance in the intracellular distribution. An equilibrium with substantial bidirectional active transport might be less favorable energetically to an ensemble of stochastic RNA granules. Conceivably, cytoplasmic RNA granules may undertake intracellular surveillance until they receive appropriate cues for anchoring/active transport. The transition between different classes of motion could thereby provide a layer of regulation in gene expression. RNA imaging with MCP-4xsfGFP is an ideal tool to image transient neuronal granules in live tissues of various animal models.

We have resolved RNAs in specific cells, which is difficult to achieve with FISH, revealing the surprising diversity of *Actb* mRNA dynamics across cell types for this ubiquitous housekeeping gene*.* Specifically, we have shown the distribution and dynamics of *Actb* RNA granules in the glial cells are remarkably heterogeneous and distinguished from those in the neurons. The substantial localization of *Actb* RNA granules in the distal processes of *Gfap+* cells, including astrocytes and radial glia-like cells, could be due to a greater need for the local synthesis of β-actin protein. The *cis*-element and *trans*-acting factors underlying *Actb* mRNA localization have been identified using in vitro fibroblasts and neurons [49–51]. The same mechanism is likely to be involved in the localization of *Actb* mRNAs in *Gfap+* cells, but questions are open regarding which specific molecules facilitate the cell-type variability. MCP-4 × sfGFP could be useful for investigating the cell-specific regulation in the native niche of brain circuits. *Gfap+* glia includes astrocytes, a major cell type accounting for approximately 30% of the cells in the mammalian CNS with many diverse functions in synaptic formation and plasticity, regulation of blood flow, and metabolic support of neighboring neurons. The distinct mRNA dynamics in astrocytes could suggest diversified function of *Actb* mRNAs in the glial cells. However, our MCP-4 × sfGFP results require additional verifications, e.g., whether immobile *Actb* RNA granules in astrocytes, which are relatively infrequent in neurons, are specific characteristics of reactive astrocytes induced by high-titer transduction with the AAV serotype (i.e., AAV5) [52]. Direct observations of dynamic RNA granules of astrocytes in live brain tissue could illuminate the function in neuro-glia interactions, such as reactive astrogliosis and neuroinflammation, and offer novel perspectives on their roles in age-related and neurodegenerative disorders.

## Methods

### Animals

All procedures were approved by the Indiana University Institutional Animal Care and Use Committee (IACUC). All Cre strains and WT mice were obtained from Jackson Laboratory: B6.Cg-Tg(Camk2a-cre)T29-1Stl/J (#005359), C57BL/6-Tg(Grik4-cre)G32-4Stl/J (#006474), B6.Cg-Tg(Gfap-cre)73.12Mvs/J (#012886), and C57BL/6J (#000664). Knock-in mouse *Actb*^*MBS/MBS*^ was a gift from R.H. Singer. To create hybrid *Cre+ Actb*^*MBS/MBS*^ mice, *Actb*^*MBS/MBS*^ was bred with Cre mice and then the offspring animals of *Actb*^*MBS/+*^*;Cre+* were bred with *Actb*^*MBS/MBS*^. *Actb*^*MBS/MBS*^*;Cre+* mice were identified in the subsequent generations. Both male and female mice in the age range of 2–5 months old were used for experiments. Animals were euthanized by CO_2_ inhalation, then cervical dislocation and decapitation. Anesthesia was done by isoflurane inhalation (2%). Analgesic drugs, Carprofen (20 mg/kg), were given subcutaneously, prior to operation and every 24 hours for 48 hours post-surgery.

### Cloning MCP constructs

A plasmid for Cre-dependent MCP-1 × GFP (pAAV-Ef1a-DIO-MCP-1 × GFP-WPRE-hGHpA) was cloned by inserting MCP into pAAV-Ef1a-DIO-EGFP-WPRE-pA (Addgene plasmid #37084) using HiFi DNA Assembly Master Mix (New England Biolabs, E2621S). A plasmid for Cre-independent MCP-1 × GFP (pAAV-Ef1a-MCP-1 × GFP-WPRE-hGHpA) was produced by inverting pAAV-Ef1a-DIO-MCP-1 × GFP-WPRE-hGHpA with Cre Recombinase (New England Biolabs, M0298S). For creating plasmids for Cre-independent and -dependent MCP-4 × sfGFP (pAAV-UbC-MCP-4xsfGFP-WPRE and pAAV-UbC-DIO-SV40NLS-MCP-4xsfGFP-W3, respectively), DNA fragments were synthesized (Genewiz) and concatenated into a pAAV backbone containing a bovine growth hormone poly(A) signal (bGHpA). To produce a plasmid for expressing MCP-4 × sfGFP under the control of GfaABC1D (pAAV-GfapABC1D-MCP-4 × sfGFP-W3-bGHpA), a fragment including the promoter was amplified by polymerase chain reaction (PCR) from pAAV.GfaABC1D.PI.Lck-GFP.SV40 (Addgene plasmid #105598) and, along with MCP-4xsfGFP, ligated into a pAAV backbone. The sequences are deposited at GenBank and the accession numbers are in S1 Table.

### AAV

AAV packaging was performed by Charles River Laboratory, Franklin Biolabs, and Vector Biolabs. Capsid serotypes of AAV2, AAV5, or AAV9 were used. Briefly, HEK293 cells were co-transfected with ultrapure plasmids and recombinant AAV particles were purified by ultrahigh speed gradient centrifugation. Genome titer was determined either by droplet digital PCR (ddPCR) or by SYBR green qPCR. All AAVs were stored in −80°C until usage. The properties of AAVs are listed in S1 Table.

### Stereotaxic injection

AAV injection was performed on the stereotaxic frame (Kopf). The coordinates for the hippocampus were AP 2.0 mm, ML 2.0 mm, DV 2.0 mm. The animal was anesthetized with 2% isoflurane, and the hair was shaved. The skin was sterilized, and a midline incision was made. The skull was drilled at the target position with a microdrill (Foredom). Using a 33-gauge needle and a Hamilton syringe, 1-µL AAV was slowly injected with at a rate of 0.1 µL/minute. After injection, the incision was closed with surgical staples.

### Acute brain slice preparation

Sectioning of fresh mouse brain was done using a vibratome (Leica) in an ice-cold artificial cerebrospinal fluid (ACSF) perfused with 95%O_2_/5%CO_2_. The thickness of slice was 250 µM. Immediately after sectioning, the brain slices were transferred to a bath containing ACSF perfused with 95%O_2_/5%CO_2_ and incubated at 37ºC for at least 1 hour for recovery.

### Pharmacology

Jasplakinolide and nocodazole (Santa Cruz Biotechnology, sc-202191 and sc-3518B, respectively) were dissolved in DMSO and added to the ACSF. Tetracaine hydrochloride (Sigma) was dissolved in water and added to the ACSF.

### Two-photon fluorescence microscopy

Two-photon microscopy was performed as described previously [37,38]. Briefly, a laser beam at 920 nm from a Ti:Sapphire laser (Chameleon Ultra, Coherent, Inc.) was used to excite GFP. The pulse duration was approximately 100 fs and the repetition rate was 80 MHz. A water-immersion microscope objective lens (HC FLUOTAR L 25x 0.95NA, Leica) was used to focus the excitation beam onto the sample. The average power at the sample was approximately 20 mW. Two detection channels employed narrow-bandpass filters (<20-nm bandwidth) and photomultiplier tubes (PMTs; H7422-40, Hamamatsu, Inc.). Images of 512 × 512 pixels were acquired at a frame rate of 0.74, 1.48, or 2.96 Hz.

### Image processing and data analysis

Image processing was performed using ImageJ [53]. RNA puncta in Fig 3 were detected using the ‘Find Maxima’ function. A prominence value was selected for NLS-MCP-4 × sfGFP that matches the visual inspection and then a quarter of the value was used for MCP-1 × GFP. Image segmentation in Fig 3(b) was obtained with an ImageJ plugin, Trainable Weka Segmentation [54], which was trained to segment the images into two classes of soma and non-soma (or neuropils). The RNA puncta were removed from the soma segment. SPT in Figs 4(d) and (e) was carried out with another ImageJ plugin, TrackMate [55], on an MCP-4 × sfGFP image of 16-bit, 360 × 360 pixels (0.08 µm/pixel). The particles were detected with a Loss-of-Gaussian detector with default parameters (a diameter of 10 pixels and a threshold of 12). The quality threshold was selected to match the spots by visual inspection. Tracking was done using default parameters (a linking max distance of 20.0 pixels, a gap-closing max distance of 15 pixels, and a gap-closing max frame gap of 2). Curve fitting for the jump distance distribution in Fig 4(e) was performed with MATLAB (MathWorks, Inc.). The equation for the probability density function p(r,t) of the two-component 2D diffusion model was $p(r,t)dr=\left(\frac{\alpha_{\text{1}}}{\text{2}D_{\text{1}}t}e^{-r^{\text{2}}/\text{4}D_{\text{1}}t}+\frac{\alpha_{\text{2}}}{\text{2}D_{\text{2}}t}e^{-r^{\text{2}}/\text{4}D_{\text{2}}t}\right)rdr$, where r is the distance, t is the time, $\alpha_{\text{1}}$ and $\alpha_{\text{2}}$ are the fractions of two components, and $D_{\text{1}}$ and $D_{\text{2}}$ are the diffusion constants.

## Supporting information

S1 Table The properties of AAVs. (DOCX)

S1 Movie *Actb*^*MBS/MBS*^ brain expressing MCP-4 × sfGFP. 30 × speed and time = 405 seconds.
(AVI)

S2 Movie MCP-4 × sfGFP expressing cells in the *Actb*^*MBS/MBS*^*;Grik4-Cre* hippocampus. 30 × speed and time = 162 seconds.
(AVI)

S3 Movie MCP-4 × sfGFP expressing *Gfap*+ cell in the *Actb*^*MBS/MBS*^ hippocampus. 30 × speed and time = 33.8 seconds.
(AVI)

S1 File Tabulated intensity values of soma and RNA shown in Fig 3(c). (CSV)

S2 File Tabulated displacement values of the jump distance distribution shown in Fig 4(e). (CSV)
