# Supplementary material for: Amplified MS2 labeling reveals heterogeneous dynamics of RNA granules in the live murine brain ex vivo
Source: PLoS One. 2026 Nov 5;21(11):e0361966. doi: 10.1371/journal.pone.0361966 (PMC13739103; doi:10.1371/journal.pone.0361966)
Supplement: Supplementary file 1 [file pone.0361966.s001.docx]

Table S1. The properties of AAVs.

|  | Vector | Capsid | Titer (GC/mL) | Source | GenBank accession number |
| --- | --- | --- | --- | --- | --- |
| 1 | Ef1a- MCP-1×GFP | AAV9 | 1.44E13 | Charles River Laboratories | PZ458741 |
| 2 | Ef1a-DIO-MCP-1×GFP | AAV2 | 7.68E12 | Charles River Laboratories | PZ458742 |
| 3 | UbC-MCP-4xsfGFP | AAV5 | 8.76E12 | Franklin Biolabs | PZ398260 |
| 4 | UbC-DIO-SV40NLS-MCP-4xsfGFP | AAV5 | 1.17E13 | Vector Biolabs | PZ398261 |
| 5 | GfapABC1D-MCP-4×sfGFP | AAV5 | 4.67E12 | Franklin Biolabs | PZ398262 |
