# Supplementary material for: Amplified MS2 labeling reveals heterogeneous dynamics of RNA granules in the live murine brain ex vivo
Source: PLoS One. 2026 Nov 5;21(11):e0361966. doi: 10.1371/journal.pone.0361966 (PMC13739103; doi:10.1371/journal.pone.0361966)
Supplement: Supplementary file 2 [file pone.0361966.s008.docx]

We would like to thank the reviewers for the thoughtful comments. They have allowed us to identify weaknesses in the previous version, and we made every effort to understand them completely. We believe all the raised concerns are addressed in the revised manuscript.

Reviewer #1 has concerns that our conclusions on tool performance, granule composition, and RNA dynamics are stronger than the current evidence allows. Specifically,

1. *… As presented, the comparison does not isolate GFP copy number as the only variable.*

As the reviewer points out, our comparisons do not isolate GFP copy number as the only variable: In Fig.1, MCP-1×GFP and MCP-4xsfGFP are driven by two separate promoters, Ef1α and UbC, respectively, which, even though being both ubiquitous, could yield different levels of expression. In Fig.3, where we sought to improve the performance of MCP-4xsfGFP, it included NLS that MCP-1×GFP didn’t. There are other differences in the two constructs being compared, e.g., poly-A signals, some of which were inevitable due to the AAV capacity. Also, two sets of AAVs were different in terms of serotype and titer. In response to this critique, we revised the manuscript to make it clear that other variables are involved as well; especially for our quantitative analysis in Fig.3, where we think NLS is more relevant than other factors in terms of the contrast for RNAs, we re-labeled two data sets as ‘MCP-1×GFP’ and ‘NLS-MCP-4xsfGFP’.

The reviewer comments that ‘… *specificity depends on controlling the amount and distribution of free MCP reporter*’. The specificity of fluorescent puncta to RNAs is tested and confirmed by negative control with WT. Ideally, free MCP reporters should be present in equilibrium with bound MCP reporters, but if there are far more MCPs than the affinity to RNA targets, then the sensitivity for RNAs could degrade. In practice, however, MS2 labeling is rarely done in a manner that can claim absolute control of free MCP reporters. It is possible that free MCP-1×GFP driven by the Ef1α promoter was relatively higher compared to MCP-4×sfGFP driven by UbC. Without a means to determine the amount of free MCPs, however, we are unable to conclude that free MCP-1×GFP’s exist beyond saturation. If they do, we anticipate that the contrast for mRNAs would be higher in some cells with relatively lower MCP expressions (as long as the sensitivity is sufficient). We did not find this to be the case. Also, the signal-to-background ratios (SBR) determined in Fig. 3 were 2.04 and 5.61 for MCP-1×GFP and MCP-4×sfGFP, respectively, which appear to approximately maintain the proportionality given the differential compositions of RNA species visualized. However, we agree that there could be also contribution from non-specific backgrounds, so we noted the possibility in the revised manuscript. We cannot compare quantitatively non-specific backgrounds between samples. Nonetheless, the soma background was used as a normalizing reference in Fig.3 because it is still appropriate for evaluating the signal to background ratios. We realize that the images in Fig.3a might suggest more abundant free MCPs in MCP-1×GFP. It is mainly because the contrasts were stretched using ImageJ’s Auto Brightness/Contrast, elevating the soma background in MCP-1×GFP roughly on par with MCP-4xsfGFP puncta. In response to this critique, we adjusted the contrast of Fig. 3a to match the intensity scales of Fig.3c, where the soma backgrounds are matched.

The reviewer suspects ‘*the more diffuse or blurred MCP-1×GFP pattern could reflect higher free reporter expression or different subcellular distribution rather than poorer labeling performance per se’.* In Fig.1, both MCP-1×GFP and MCP-4xsfGFP were without an NLS, so subcellular distribution doesn’t explain the observed puncta. NLS-MCP-1×GFP was not used for our Fig.3, but it has been in the literature (e.g., Ref. 35 of the revised manuscript) and *Actb* mRNAs in the hippocampal slices visualized by NLS-MCP-1×GFP (Fig.2D, Ref. 35) look remarkably different from our data of NLS-MCP-4xsfGFP. Taken together, it is clear that MCP-4xsfGFP achieves significantly higher sensitivity than MCP-1×GFP, regardless of the inclusion of NLS.

2. … *the central claim that MCP-4×sfGFP reveals a broader and more representative population of RNA granules is not sufficiently validated.*

The statement that 13 times more puncta by MCP-4×sfGFP can reveal a broader and more representative population of RNA granules than MCP-1×GFP does is hardly hyperbolic. However, we understand that the reviewer’s concern is more regarding uniform representation across the subpopulations of mRNAs. Given the lack of orthogonal validations, we revised the conclusion on granule composition to indicate the presence of potential biases. In principle, this information can be obtained through comparison against the ground truth of subpopulations of RNPs. However, we are skeptical about the practicality of smFISH providing an appropriate benchmark for MCP-4xsfGFP, as the reviewer suggests, on several accounts. First, smFISH data could capture the ground truth of mRNA populations only if the efficiency of detection is near unity, which is nontrivial to verify and requires thorough validation of its own. For example, in one of the best results of the literature, Raj et al. found 81% and 85% of FISH signals targeting the same mRNAs in CHO cells overlap (PMID: 18806792). The efficiency would be substantially lower for tissues. Second, the fluorescence signals from MCP-4xsfGFP are unlikely to maintain the proportionality before and after tissue processing. Fixation will alter the optical properties of MCP-4xsfGFP as well as the embedding tissue, confounding the co-registration with any fixed tissue labeling.

We are puzzled by the reviewer’s comment that ‘…*the inference that intensity subpopulations … remains indirect and model-dependent’*, as our analysis of intensity subpopulations is largely identical to that of quantitative smFISH (e.g., PMID: 22301778, PMID: 9554849). A major difference is that unit fluorescence in our analysis comes from the mode spacing instead of single mRNA molecules. The reviewer thinks that the inference is *model*-dependent. We wonder if it is possible that the reviewer misunderstood the use of Gaussian mixture model, the only ‘model’ employed in our paper, for fitting the intensity data, in which the discrete peaks are already identifiable. Each Gaussian has **mean**, covariance and weight as free parameters. The number of Gaussians was determined by the maximum likelihood, i.e., one that minimizes the Akaike information criteria value. As a result, the outcome that discrete modes are quantal is not pre-programmed nor likely an artifact of the model. To address this critique, we provided more details on our Gaussian mixture model.

3. *… some biological interpretations are overstated. The conclusion that neuronal Actb granule movement is largely independent of active transport…* *The glial-cell conclusions are also preliminary…*

We repeated each pharmacological experiment two or three times. We did not acquire data for quantitative analysis because we anticipated the effects of drugs, if there is any, would halt the motions immediately and evidently. In response to these comments, the mentioned biological interpretations and conclusions are revised/moved/consolidated into Discussion.

4. … *it does not provide the full construct sequences… the image analysis workflow is underdescribed…*

The sequences of all AAV constructs have been submitted to GenBank and the accession numbers are provided (Table S1). Also, the manuscript is revised to provide details on image processing, including segmentation training, tracking parameters, inclusion criteria, or the underlying quantitative datasets (as required to replicate plots in Fig. 3 and Fig. 4).

Reviewer #2 recommends a number of improvements. Specifically,

1. *In the spot detection analysis (Figure 3b), some spots were detected in the MCP-1xGFP image. However, the fluorescence was nearly homogeneous within each cell, with few obvious spots in the original image (Figure 3a). This suggests that the parameters for spot detection, especially the “Quality” threshold in TrackMate, were not set correctly.*

The reviewer incorrectly thinks that spot detection in the MCP-1xGFP image of Fig. 3 was performed using TrackMate. Separate methods of spot detection were used in Figs. 3 and 4 for the signal-to-background ratio (SBR) analysis and single-particle tracking (SPT), respectively. For spots in Fig. 3, the ‘Find Maxima’ function in ImageJ was used, while for SPT in Fig.4, TrackMate was used. SPT was not performed for MCP-1×GFP. In response to this critique, we included information on the parameters used for spot detection in Figs. 3 and 4.

2. *Considering the number of sfGFP molecules in a single RNA granule spot, the fluorescent intensity distribution of detected particles will be continuous rather than discrete.* … *the rationale of this fitting and the presence of multiple components should be explained.*

We revised to clarify that images in Fig.3(a) are max projections of 12-µm z-stacks. It reduces the variability due to defocus. The fluorescence would be continuous, as the reviewer suggests, if the variations in the number of MCPs bound to RNA are much greater than the discreteness, or 48 per RNA. Even though the variations in the number of bound MCPs are likely to be the same for MCP-1xGFP and MCP-4xsfGFP, the amplified discreteness relative to the background could make it easier to resolve the number of RNAs within granules. Gaussian mixture model (GMM) is a standard statistical analysis routinely employed for quantal analysis, i.e., determining the properties of multiple species in a population. To address this issue, we provided rationales for the use of GMM.

3. *The authors should explain the definition of the magenta and green spots in Figure 3c. Also, the definition of the horizontal axis in Figure 3d should be described.*

To address this critique, we added labels for the symbols in Fig. 3c and the horizontal axis in Fig. 3d.

Journal requirements:

1. Style Requirements: The manuscript is revised to meet the requirement.
2. Animal Methods Section: The manuscript is revised to include the description of animal euthanasia, anesthesia, and analgesics, and efforts to alleviate suffering.
3. Code sharing: N/A
4. Funding information: Funding information is removed from the text.
5. Grant information: The correct grant number is provided in the ‘Funding Information’.
6. Financial disclosure: The statement is included regarding the role of the funder in the cover letter: "The funders had no role in study design, data collection and analysis, decision to publish, or preparation of the manuscript."
7. Data Availability: Raw data are submitted as required to replicate plots in Fig. 3 and Fig. 4.
8. Ethics statement: N/A
9. Captions for Supporting Information: Captions for Supporting Information are included.
10. Citations: N/A
