# Supplementary material for: Amplified MS2 labeling reveals heterogeneous dynamics of RNA granules in the live murine brain ex vivo
Source: PLoS One. 2026 Nov 5;21(11):e0361966. doi: 10.1371/journal.pone.0361966 (PMC13739103; doi:10.1371/journal.pone.0361966)
Supplement: Supplementary file 3 [file pone.0361966.s009.docx]

We are grateful for the additional comments by the reviewers. We made every effort to address them as thoroughly as possible in the following.

The Editor suggested the abstract to be revised, reminding us of PLOS ONE’s editorial criteria. We made changes across the manuscript in response to this recommendation (e.g., line:31-36).

Reviewer #2 raised following two concerns that still remain even after the first revision.

1. *The left panel of Figure 3c shows that there is substantially no difference between the intensity of soma and detected RNA, indicating that RNA granules were not detected with the MCP-1xGFP probe.*

As criteria, ImageJ’s Find Max uses prominence, i.e., intensity above surrounding pixel values (not the noise level as the reviewer suggests), so weak spots comparable to soma background could be detected provided that they are situated within relatively dim regions. However, as the reviewer points out, the spots by MCP-1xGFP are much more ambiguous compared to those by MCP-4xsfGFP. In response to this comment, we now state that the confidence for positive identification as RNA granules is significantly poorer due to lower contrasts (line 137-141).

2. *To evaluate whether the GMM analysis yields appropriate results and to perform the analysis correctly, it is necessary to examine the fluorescence intensity distribution of single Actb-MBS molecules, which provide a standard for GMM analysis.*

We agree with the reviewer that the uniform discrete spacings between Gaussian peaks do not necessarily prove that they are due to integer mRNA copy numbers and such association would be valid only with a standard of single mRNA molecules. We revised to emphasize this limitation. As a prior art, we include two additional references (Refs. 24 and 25) where the GMM analysis is applied to smFISH images for determining mRNA copy numbers without independent standards, based on an assumption that the cytoplasmic signals represent the intensity of single copy, and how our images are different (line: 158-164).

Reviewer #3 had the following critiques.

1. *Therefore, the improved performance cannot yet be clearly attributed to the 4×sfGFP itself.*

In response to this comment, we now state that there could be other contributing factors for the improved performance (line: 142-144).

2. *The GMM analysis identifies statistical subpopulations but does not directly demonstrate that these populations represent different RNA copy numbers.*

This comment is also echoed by Reviewer #2, and we address it as above.

3. *The conclusion in Figure 4 that RNA granule movement is “independent of active transport” appears too strong. …* *no obvious effect was detected under the current experimental conditions.*

In response to this suggestion, we removed the phrase from the section title for Figure 4 (line 165) and revised relevant texts (line: 239-240) and added the suggested edit regarding the limitation of the current experimental conditions (line 174-175). It is described in more detail in a paragraph in the Discussion (line 244-249).

4. *The authors should carefully check the figure order and formatting before publication.*

The formatting of submitted manuscript was done automatically by the PLOS ONE system. We will double check the figure order during resubmission.
