# Supplementary material for: Amplified MS2 labeling reveals heterogeneous dynamics of RNA granules in the live murine brain ex vivo
Source: PLoS One. 2026 Nov 5;21(11):e0361966. doi: 10.1371/journal.pone.0361966 (PMC13739103; doi:10.1371/journal.pone.0361966)
Supplement: Peer review history

# Peer review: Decision Letter 0

**DOI:** 10.1371/journal.pone.0361966.r001

**Authors:** Mohsan Ullah

27 Apr 2026

Dear Dr.  Lim,

Thank you for submitting your manuscript to PLOS ONE. After careful consideration, we feel that it has merit but does not fully meet PLOS ONE’s publication criteria as it currently stands. Therefore, we invite you to submit a revised version of the manuscript that addresses the points raised during the review process.

Please submit your revised manuscript by Jun 11 2026 11:59PM. If you will need more time than this to complete your revisions, please reply to this message or contact the journal office at plosone@plos.org. When you're ready to submit your revision, log on to https://www.editorialmanager.com/pone/ and select the 'Submissions Needing Revision' folder to locate your manuscript file.

- A letter that responds to each point raised by the academic editor and reviewer(s). You should upload this letter as a separate file labeled 'Response to Reviewers'.
- A marked-up copy of your manuscript that highlights changes made to the original version. You should upload this as a separate file labeled 'Revised Manuscript with Track Changes'.
- An unmarked version of your revised paper without tracked changes. You should upload this as a separate file labeled 'Manuscript'.

If you would like to make changes to your financial disclosure, please include your updated statement in your cover letter. Guidelines for resubmitting your figure files are available below the reviewer comments at the end of this letter.

If applicable, we recommend that you deposit your laboratory protocols in protocols.io to enhance the reproducibility of your results. Protocols.io assigns your protocol its own identifier (DOI) so that it can be cited independently in the future. For instructions see: https://journals.plos.org/plosone/s/submission-guidelines#loc-laboratory-protocols. Additionally, PLOS ONE offers an option for publishing peer-reviewed Lab Protocol articles, which describe protocols hosted on protocols.io. Read more information on sharing protocols at https://plos.org/protocols?utm_medium=editorial-email&utm_source=authorletters&utm_campaign=protocols.

As the corresponding author, your ORCID iD is verified in the submission system and will appear in the published article. PLOS supports the use of ORCID, and we encourage all coauthors to register for an ORCID iD and use it as well. Please encourage your coauthors to verify their ORCID iD within the submission system before final acceptance, as unverified ORCID iDs will not appear in the published article. *Only*  the individual author can complete the verification step; PLOS staff *cannot*  verify ORCID iDs on behalf of authors.

We look forward to receiving your revised manuscript.

Kind regards,

Mohsan Ullah, Ph.D.

Academic Editor

PLOS One

Journal Requirements:

When submitting your revision, we need you to address these additional requirements.

1.Please ensure that your manuscript meets PLOS ONE's style requirements, including those for file naming. The PLOS ONE style templates can be found at

https://journals.plos.org/plosone/s/file?id=wjVg/PLOSOne_formatting_sample_main_body.pdf and

https://journals.plos.org/plosone/s/file?id=ba62/PLOSOne_formatting_sample_title_authors_affiliations.pdf

2. To comply with PLOS One submissions requirements, in your Methods section, please provide additional information regarding the experiments involving animals and ensure you have included details on (1) methods of sacrifice, (2) methods of anesthesia and/or analgesia, and (3) efforts to alleviate suffering.

3. Please note that PLOS One has specific guidelines on code sharing for submissions in which author-generated code underpins the findings in the manuscript. In these cases, we expect all author-generated code to be made available without restrictions upon publication of the work. Please review our guidelines at https://journals.plos.org/plosone/s/materials-and-software-sharing#loc-sharing-code and ensure that your code is shared in a way that follows best practice and facilitates reproducibility and reuse.

4. Please note that funding information should not appear in any section or other areas of your manuscript. We will only publish funding information present in the Funding Statement section of the online submission form. Please remove any funding-related text from the manuscript.

5. We note that the grant information you provided in the ‘Funding Information’ and ‘Financial Disclosure’ sections do not match.

When you resubmit, please ensure that you provide the correct grant numbers for the awards you received for your study in the ‘Funding Information’ section.

6. Thank you for stating the following financial disclosure:

“NIH GM140841”

Please state what role the funders took in the study.  If the funders had no role, please state: "The funders had no role in study design, data collection and analysis, decision to publish, or preparation of the manuscript."

If this statement is not correct you must amend it as needed.

Please include this amended Role of Funder statement in your cover letter; we will change the online submission form on your behalf.

7. We note that your Data Availability Statement is currently as follows: All relevant data are within the manuscript and in Supporting Information files.

Please confirm at this time whether or not your submission contains all raw data required to replicate the results of your study. Authors must share the “minimal data set” for their submission. PLOS defines the minimal data set to consist of the data required to replicate all study findings reported in the article, as well as related metadata and methods (https://journals.plos.org/plosone/s/data-availability#loc-minimal-data-set-definition).

For example, authors should submit the following data:

- The values behind the means, standard deviations and other measures reported;

- The values used to build graphs;

- The points extracted from images for analysis.

Authors do not need to submit their entire data set if only a portion of the data was used in the reported study.

If your submission does not contain these data, please either upload them as Supporting Information files or deposit them to a stable, public repository and provide us with the relevant URLs, DOIs, or accession numbers. For a list of recommended repositories, please see https://journals.plos.org/plosone/s/recommended-repositories.

If there are ethical or legal restrictions on sharing a de-identified data set, please explain them in detail (e.g., data contain potentially sensitive information, data are owned by a third-party organization, etc.) and who has imposed them (e.g., an ethics committee). Please also provide contact information for a data access committee, ethics committee, or other institutional body to which data requests may be sent. If data are owned by a third party, please indicate how others may request data access.

8. Please include your full ethics statement in the ‘Methods’ section of your manuscript file. In your statement, please include the full name of the IRB or ethics committee who approved or waived your study, as well as whether or not you obtained informed written or verbal consent. If consent was waived for your study, please include this information in your statement as well.

9.Please include captions for your Supporting Information files at the end of your manuscript, and update any in-text citations to match accordingly. Please see our Supporting Information guidelines for more information: http://journals.plos.org/plosone/s/supporting-information.

10. If the reviewer comments include a recommendation to cite specific previously published works, please review and evaluate these publications to determine whether they are relevant and should be cited. There is no requirement to cite these works unless the editor has indicated otherwise.

[Note: HTML markup is below. Please do not edit.]

Reviewers' comments:

Reviewer's Responses to Questions

**Comments to the Author**

1. Is the manuscript technically sound, and do the data support the conclusions?

Reviewer #1: Partly

Reviewer #2: Yes

**********

2. Has the statistical analysis been performed appropriately and rigorously?

Reviewer #1: N/A

Reviewer #2: N/A

**********

3. Have the authors made all data underlying the findings in their manuscript fully available?

The PLOS Data policy

Reviewer #1: No

Reviewer #2: Yes

**********

4. Is the manuscript presented in an intelligible fashion and written in standard English?

Reviewer #1: Yes

Reviewer #2: Yes

**********

Reviewer #1: This manuscript presents a potentially useful imaging approach for visualizing Actb RNA granules in acute murine brain slices using MCP-4×sfGFP. The technical goal is clear, and the study includes relevant negative controls in WT mice lacking MBS. However, under PLOS ONE criteria, the main issue is whether the data rigorously support the claims. In my view, they do not yet do so consistently. Several key conclusions about tool performance, granule composition, and RNA dynamics are stronger than the current evidence allows.

My first major concern is insufficient validation of the labeling tool itself. Because GFP fluorescence is independent of RNA binding, specificity depends on controlling the amount and distribution of free MCP reporter. The manuscript does not quantify reporter expression or assess whether unbound reporter could account for background and apparent differences between constructs. Therefore, the more diffuse or blurred MCP-1×GFP pattern could reflect higher free reporter expression or different subcellular distribution rather than poorer labeling performance per se. As presented, the comparison does not isolate GFP copy number as the only variable.

Second, the central claim that MCP-4×sfGFP reveals a broader and more representative population of RNA granules is not sufficiently validated. The evidence is based mainly on increased puncta counts and higher relative intensity compared with MCP-1×GFP, but there is no orthogonal benchmark, such as smFISH or fixed-tissue validation, to show how complete or unbiased this detection is. Similarly, the inference that intensity subpopulations reflect different mRNA copy numbers within granules is plausible but remains indirect and model-dependent.

Third, some biological interpretations are overstated. The conclusion that neuronal Actb granule movement is largely independent of active transport is weakened by the authors’ own acknowledgement that the imaging frame rate was only about 1 Hz and may miss brief directional transport events. The pharmacological experiments are also described largely qualitatively, with limited detail on sample size and quantitative support. The glial-cell conclusions are also preliminary, given the acknowledged issues with Gfap-Cre specificity and the possibility that AAV5 transduction may induce reactive astrocyte states that alter granule behavior.

Fourth, reproducibility is currently inadequate. Although the manuscript describes how the constructs were assembled, it does not provide the full construct sequences for others to reproduce the work. More broadly, the image analysis workflow is underdescribed: the manuscript names ImageJ, Trainable Weka Segmentation, TrackMate, and MATLAB, but does not provide enough detail on segmentation training, tracking parameters, inclusion criteria, or the underlying quantitative datasets. The generic data availability statement also does not make clear whether the minimal reusable dataset has actually been provided.

Overall, I find the approach promising, but the present manuscript does not yet provide sufficiently rigorous validation to support its main claims at the level required for publication. The study would be stronger with direct validation of reporter specificity and expression, a fairer matched comparison between the 1× and 4× reporters, stronger orthogonal validation of detection performance, fuller quantitative support for the motion analyses, and complete construct sequence information to ensure reproducibility.

Reviewer #2: This article describes an improvement to the MS2 method for monitoring RNA granules in brain slice samples, achieving higher sensitivity than the previous approach. In this improvement, the authors created an RNA labeling tool with a four-tandem repeat of super-folder GFP (sfGFP) fused to the MS2-binding protein (MBP), which yields higher detectability of RNA granules than a single GFP. Using the tool, RNA granules in brain slice samples were monitored, and the properties of the detected granules, such as intensity and motility, were analyzed and compared with results from the conventional MS2 tool.

The detectability of RNA granules was highly improved by using the developed probe compared to the conventional one with a single GFP, and therefore, the tool may become a convenient tool for the analysis of RNA in slice samples. For a more precise characterization of the probe, some improvements to the granule analysis are recommended.

1. In the spot detection analysis (Figure 3b), some spots were detected in the MCP-1xGFP image. However, the fluorescence was nearly homogeneous within each cell, with few obvious spots in the original image (Figure 3a). This suggests that the parameters for spot detection, especially the “Quality” threshold in TrackMate, were not set correctly. The accuracy of spot detection would affect the results of the motility analysis of RNA granules shown in Figure 4e and 4d. To identify appropriate parameters, the reviewer recommends conducting spot detection on images of WT samples expressing MCP-4xsfGFP that do not include RNA granules labeled with the probe, and then determining the appropriate parameters. Also, the reviewer recommends that the authors describe the TrackMate analysis parameters in the Materials and Methods section.

2. Considering the number of sfGFP molecules in a single RNA granule spot, the fluorescent intensity distribution of detected particles will be continuous rather than discrete. Although the authors performed multiple Gaussian fits to the intensity histogram, the theoretical basis for this approach is unclear. If the authors retain this analysis, the rationale of this fitting and the presence of multiple components should be explained.

3. Minor comment. The authors should explain the definition of the magenta and green spots in Figure 3c. Also, the definition of the horizontal axis in Figure 3d should be described.

**********

what does this mean?). If published, this will include your full peer review and any attached files.

If you choose “no”, your identity will remain anonymous but your review may still be made public.

**Do you want your identity to be public for this peer review?**  For information about this choice, including consent withdrawal, please see our Privacy Policy

Reviewer #1: No

Reviewer #2: No

**********

[NOTE: If reviewer comments were submitted as an attachment file, they will be attached to this email and accessible via the submission site. Please log into your account, locate the manuscript record, and check for the action link "View Attachments". If this link does not appear, there are no attachment files.]

To ensure your figures meet our technical requirements, please review our figure guidelines: https://journals.plos.org/plosone/s/figures

You may also use PLOS’s free figure tool, NAAS, to help you prepare publication quality figures: https://journals.plos.org/plosone/s/figures#loc-tools-for-figure-preparation.

NAAS will assess whether your figures meet our technical requirements by comparing each figure against our figure specifications.

# Author comment: Author response to Decision Letter 1

**DOI:** 10.1371/journal.pone.0361966.r002

8 Jun 2026

We would like to thank the reviewers for the thoughtful comments. They have allowed us to identify weaknesses in the previous version, and we made every effort to understand them completely. We believe all the raised concerns are addressed in the revised manuscript.

Reviewer #1 has concerns that our conclusions on tool performance, granule composition, and RNA dynamics are stronger than the current evidence allows. Specifically,

1. … As presented, the comparison does not isolate GFP copy number as the only variable.

As the reviewer points out, our comparisons do not isolate GFP copy number as the only variable: In Fig.1, MCP-1×GFP and MCP-4xsfGFP are driven by two separate promoters, Ef1α and UbC, respectively, which, even though being both ubiquitous, could yield different levels of expression. In Fig.3, where we sought to improve the performance of MCP-4xsfGFP, it included NLS that MCP-1×GFP didn’t. There are other differences in the two constructs being compared, e.g., poly-A signals, some of which were inevitable due to the AAV capacity. Also, two sets of AAVs were different in terms of serotype and titer. In response to this critique, we revised the manuscript to make it clear that other variables are involved as well; especially for our quantitative analysis in Fig.3, where we think NLS is more relevant than other factors in terms of the contrast for RNAs, we re-labeled two data sets as ‘MCP-1×GFP’ and ‘NLS-MCP-4xsfGFP’.

The reviewer comments that ‘… specificity depends on controlling the amount and distribution of free MCP reporter’. The specificity of fluorescent puncta to RNAs is tested and confirmed by negative control with WT. Ideally, free MCP reporters should be present in equilibrium with bound MCP reporters, but if there are far more MCPs than the affinity to RNA targets, then the sensitivity for RNAs could degrade. In practice, however, MS2 labeling is rarely done in a manner that can claim absolute control of free MCP reporters. It is possible that free MCP-1×GFP driven by the Ef1α promoter was relatively higher compared to MCP-4×sfGFP driven by UbC. Without a means to determine the amount of free MCPs, however, we are unable to conclude that free MCP-1×GFP’s exist beyond saturation. If they do, we anticipate that the contrast for mRNAs would be higher in some cells with relatively lower MCP expressions (as long as the sensitivity is sufficient). We did not find this to be the case. Also, the signal-to-background ratios (SBR) determined in Fig. 3 were 2.04 and 5.61 for MCP-1×GFP and MCP-4×sfGFP, respectively, which appear to approximately maintain the proportionality given the differential compositions of RNA species visualized. However, we agree that there could be also contribution from non-specific backgrounds, so we noted the possibility in the revised manuscript. We cannot compare quantitatively non-specific backgrounds between samples. Nonetheless, the soma background was used as a normalizing reference in Fig.3 because it is still appropriate for evaluating the signal to background ratios. We realize that the images in Fig.3a might suggest more abundant free MCPs in MCP-1×GFP. It is mainly because the contrasts were stretched using ImageJ’s Auto Brightness/Contrast, elevating the soma background in MCP-1×GFP roughly on par with MCP-4xsfGFP puncta. In response to this critique, we adjusted the contrast of Fig. 3a to match the intensity scales of Fig.3c, where the soma backgrounds are matched.

The reviewer suspects ‘the more diffuse or blurred MCP-1×GFP pattern could reflect higher free reporter expression or different subcellular distribution rather than poorer labeling performance per se’. In Fig.1, both MCP-1×GFP and MCP-4xsfGFP were without an NLS, so subcellular distribution doesn’t explain the observed puncta. NLS-MCP-1×GFP was not used for our Fig.3, but it has been in the literature (e.g., Ref. 35 of the revised manuscript) and Actb mRNAs in the hippocampal slices visualized by NLS-MCP-1×GFP (Fig.2D, Ref. 35) look remarkably different from our data of NLS-MCP-4xsfGFP. Taken together, it is clear that MCP-4xsfGFP achieves significantly higher sensitivity than MCP-1×GFP, regardless of the inclusion of NLS.

2. … the central claim that MCP-4×sfGFP reveals a broader and more representative population of RNA granules is not sufficiently validated.

The statement that 13 times more puncta by MCP-4×sfGFP can reveal a broader and more representative population of RNA granules than MCP-1×GFP does is hardly hyperbolic. However, we understand that the reviewer’s concern is more regarding uniform representation across the subpopulations of mRNAs. Given the lack of orthogonal validations, we revised the conclusion on granule composition to indicate the presence of potential biases. In principle, this information can be obtained through comparison against the ground truth of subpopulations of RNPs. However, we are skeptical about the practicality of smFISH providing an appropriate benchmark for MCP-4xsfGFP, as the reviewer suggests, on several accounts. First, smFISH data could capture the ground truth of mRNA populations only if the efficiency of detection is near unity, which is nontrivial to verify and requires thorough validation of its own. For example, in one of the best results of the literature, Raj et al. found 81% and 85% of FISH signals targeting the same mRNAs in CHO cells overlap (PMID: 18806792). The efficiency would be substantially lower for tissues. Second, the fluorescence signals from MCP-4xsfGFP are unlikely to maintain the proportionality before and after tissue processing. Fixation will alter the optical properties of MCP-4xsfGFP as well as the embedding tissue, confounding the co-registration with any fixed tissue labeling.

We are puzzled by the reviewer’s comment that ‘…the inference that intensity subpopulations … remains indirect and model-dependent’, as our analysis of intensity subpopulations is largely identical to that of quantitative smFISH (e.g., PMID: 22301778, PMID: 9554849). A major difference is that unit fluorescence in our analysis comes from the mode spacing instead of single mRNA molecules. The reviewer thinks that the inference is model-dependent. We wonder if it is possible that the reviewer misunderstood the use of Gaussian mixture model, the only ‘model’ employed in our paper, for fitting the intensity data, in which the discrete peaks are already identifiable. Each Gaussian has mean, covariance and weight as free parameters. The number of Gaussians was determined by the maximum likelihood, i.e., one that minimizes the Akaike information criteria value. As a result, the outcome that discrete modes are quantal is not pre-programmed nor likely an artifact of the model. To address this critique, we provided more details on our Gaussian mixture model.

3. … some biological interpretations are overstated. The conclusion that neuronal Actb granule movement is largely independent of active transport… The glial-cell conclusions are also preliminary…

We repeated each pharmacological experiment two or three times. We did not acquire data for quantitative analysis because we anticipated the effects of drugs, if there is any, would halt the motions immediately and evidently. In response to these comments, the mentioned biological interpretations and conclusions are revised/moved/consolidated into Discussion.

4. … it does not provide the full construct sequences… the image analysis workflow is underdescribed…

The sequences of all AAV constructs have been submitted to GenBank and the accession numbers are provided (Table S1). Also, the manuscript is revised to provide details on image processing, including segmentation training, tracking parameters, inclusion criteria, or the underlying quantitative datasets (as required to replicate plots in Fig. 3 and Fig. 4).

Reviewer #2 recommends a number of improvements. Specifically,

1. In the spot detection analysis (Figure 3b), some spots were detected in the MCP-1xGFP image. However, the fluorescence was nearly homogeneous within each cell, with few obvious spots in the original image (Figure 3a). This suggests that the parameters for spot detection, especially the “Quality” threshold in TrackMate, were not set correctly.

The reviewer incorrectly thinks that spot detection in the MCP-1xGFP image of Fig. 3 was performed using TrackMate. Separate methods of spot detection were used in Figs. 3 and 4 for the signal-to-background ratio (SBR) analysis and single-particle tracking (SPT), respectively. For spots in Fig. 3, the ‘Find Maxima’ function in ImageJ was used, while for SPT in Fig.4, TrackMate was used. SPT was not performed for MCP-1×GFP. In response to this critique, we included information on the parameters used for spot detection in Figs. 3 and 4.

2. Considering the number of sfGFP molecules in a single RNA granule spot, the fluorescent intensity distribution of detected particles will be continuous rather than discrete. … the rationale of this fitting and the presence of multiple components should be explained.

We revised to clarify that images in Fig.3(a) are max projections of 12-µm z-stacks. It reduces the variability due to defocus. The fluorescence would be continuous, as the reviewer suggests, if the variations in the number of MCPs bound to RNA are much greater than the discreteness, or 48 per RNA. Even though the variations in the number of bound MCPs are likely to be the same for MCP-1xGFP and MCP-4xsfGFP, the amplified discreteness relative to the background could make it easier to resolve the number of RNAs within granules. Gaussian mixture model (GMM) is a standard statistical analysis routinely employed for quantal analysis, i.e., determining the properties of multiple species in a population. To address this issue, we provided rationales for the use of GMM.

3. The authors should explain the definition of the magenta and green spots in Figure 3c. Also, the definition of the horizontal axis in Figure 3d should be described.

To address this critique, we added labels for the symbols in Fig. 3c and the horizontal axis in Fig. 3d.

Attachment Submitted filename: Response to Reviewers_LimCooper.docx

# Peer review: Decision Letter 1

**DOI:** 10.1371/journal.pone.0361966.r003

**Authors:** Yu-Hsuan Tsai

18 Aug 2026

Dear Dr. Lim,

Thank you for submitting your manuscript to PLOS One. After careful consideration, we feel that it has merit but does not fully meet PLOS One’s publication criteria as it currently stands. Therefore, we invite you to submit a revised version of the manuscript that addresses the points raised during the review process.

Please submit your revised manuscript by Oct 02 2026 11:59PM. If you will need more time than this to complete your revisions, please reply to this message or contact the journal office at plosone@plos.org. When you're ready to submit your revision, log on to https://www.editorialmanager.com/pone/ and select the 'Submissions Needing Revision' folder to locate your manuscript file.

- A letter that responds to each point raised by the academic editor and reviewer(s). You should upload this letter as a separate file labeled 'Response to Reviewers'.
- A marked-up copy of your manuscript that highlights changes made to the original version. You should upload this as a separate file labeled 'Revised Manuscript with Track Changes'.
- An unmarked version of your revised paper without tracked changes. You should upload this as a separate file labeled 'Manuscript'.

If you would like to make changes to your financial disclosure, please include your updated statement in your cover letter. Guidelines for resubmitting your figure files are available below the reviewer comments at the end of this letter.

If applicable, we recommend that you deposit your laboratory protocols in protocols.io to enhance the reproducibility of your results. Protocols.io assigns your protocol its own identifier (DOI) so that it can be cited independently in the future. For instructions see: https://journals.plos.org/plosone/s/submission-guidelines#loc-laboratory-protocols. Additionally, PLOS One offers an option for publishing peer-reviewed Lab Protocol articles, which describe protocols hosted on protocols.io. Read more information on sharing protocols at https://plos.org/protocols?utm_medium=editorial-email&utm_source=authorletters&utm_campaign=protocols.

As the corresponding author, your ORCID iD is verified in the submission system and will appear in the published article. PLOS supports the use of ORCID, and we encourage all coauthors to register for an ORCID iD and use it as well. Please encourage your coauthors to verify their ORCID iD within the submission system before final acceptance, as unverified ORCID iDs will not appear in the published article. *Only*  the individual author can complete the verification step; PLOS staff *cannot*  verify ORCID iDs on behalf of authors.

We look forward to receiving your revised manuscript.

Kind regards,

Yu-Hsuan Tsai

Academic Editor

PLOS One

Journal Requirements:

If the reviewer comments include a recommendation to cite specific previously published works, please review and evaluate these publications to determine whether they are relevant and should be cited. There is no requirement to cite these works unless the editor has indicated otherwise.

Please review your reference list to ensure that it is complete and correct. If you have cited papers that have been retracted, please include the rationale for doing so in the manuscript text, or remove these references and replace them with relevant current references. Any changes to the reference list should be mentioned in the rebuttal letter that accompanies your revised manuscript. If you need to cite a retracted article, indicate the article’s retracted status in the References list and also include a citation and full reference for the retraction notice.

Additional Editor Comments:

Securing reviewers for this manuscript has proven quite difficult; unfortunately, all of the reviewers you suggested declined the invitation to review. One potential reviewer, in explaining their decision, raised a concern that I believe would be valuable for you to address before we can move forward: they felt the abstract framed the central contribution primarily around novelty and significance, for example, describing the method as enabling visualization of RNA granules in native CNS tissue for the first time, without providing sufficient technical detail to substantiate that framing. I want to clarify that under PLOS ONE's editorial criteria, novelty and perceived significance are not requirements for publication; our evaluation rests solely on the technical soundness of the experimental design, the rigor of the data, and whether the conclusions are fully supported by the evidence presented. Please revise the abstract (and, where relevant, the framing in the introduction and discussion) to foreground the technical validity and methodological details of your approach, such as the design and performance characteristics of the MCP-4×sfGFP labeling strategy, rather than emphasizing novelty claims that are not needed.

[Note: HTML markup is below. Please do not edit.]

Reviewers' comments:

Reviewer's Responses to Questions

**Comments to the Author**

Reviewer #2: (No Response)

Reviewer #3: (No Response)

**********

2. Is the manuscript technically sound, and do the data support the conclusions?

Reviewer #2: Partly

Reviewer #3: (No Response)

**********

3. Has the statistical analysis been performed appropriately and rigorously?

Reviewer #2: N/A

Reviewer #3: (No Response)

**********

4. Have the authors made all data underlying the findings in their manuscript fully available?

The PLOS Data policy

Reviewer #2: Yes

Reviewer #3: (No Response)

**********

5. Is the manuscript presented in an intelligible fashion and written in standard English?

Reviewer #2: Yes

Reviewer #3: (No Response)

**********

Reviewer #2: The manuscript has been revised based on the reviewers' comments. Regarding the responses to Reviewer 2's comments, comment 3 has been addressed appropriately. However, some points regarding the response to Comment 1 need to be verified, and there are still issues with the response to Comment 2. The following are the comments on the revised manuscript and the response letter.

1. Regarding the response to the previous Comment 1, the reviewer understands that panels in Figure 3a are maximum projections of z-stacked images, and the spot detection in Figure 3b was conducted with the Find Maxima function in ImageJ. Given the properties of the Find Maxima function, intensity peaks that exceed the noise level are automatically detected; therefore, the detected spots do not necessarily indicate the labeled RNA granules. The left panel of Figure 3c shows that there is substantially no difference between the intensity of soma and detected RNA, indicating that RNA granules were not detected with the MCP-1xGFP probe. However, the manuscript was written on the assumption that the detected points indicated the RNA. If this understanding is correct, the manuscript's expression should be improved accordingly.

2. Regarding the response to the previous Comment 2, GMM is indeed a standard method for quantal analysis, as the authors mentioned in the response letter. However, GMM can be applied to any data and produces a fit, whether appropriate or not. In the case of the RNA imaging using NLS-MCP-4xsfGFP, not all MBS were necessarily occupied by the probes, nor did all sfGFP necessarily exhibit fluorescence due to photobleaching or a folding problem. To evaluate whether the GMM analysis yields appropriate results and to perform the analysis correctly, it is necessary to examine the fluorescence intensity distribution of single Actb-MBS molecules, which provide a standard for GMM analysis.

Reviewer #3: The authors have carefully addressed the previous reviewers’ comments and have made substantial revisions to the manuscript. In particular, they have provided additional information on the experimental constructs, image-analysis methods, and relevant data, and have appropriately toned down some of the stronger biological interpretations. Overall, the revised manuscript has been improved in terms of methodological description, interpretation of the results, and reproducibility.

Remaining concerns:

1. The comparison between NLS-MCP-4xsfGFP and MCP-1xGFP remains confounded by differences in promoter, NLS, AAV vector, and viral titer. Therefore, the improved performance cannot yet be clearly attributed to the 4×sfGFP itself. The authors should discuss this limitation more explicitly.

2. In Figure 3, the correspondence between the discrete fluorescence-intensity peaks and Actb mRNA copy numbers is indirect and lacks independent validation or relevant discussion. The GMM analysis identifies statistical subpopulations, but does not directly demonstrate that these populations represent different RNA copy numbers.

3. The conclusion in Figure 4 that RNA granule movement is “independent of active transport” appears too strong. The pharmacological experiments were performed with only 2–3 replicates, and the temporal resolution of the imaging is more appropriate for concluding that no obvious effect was detected under the current experimental conditions.

4. There are also some presentation issues that should be corrected. In particular, the plasmid schematic/text in Figure 2a appears to be displayed in the wrong orientation, and Figure 3 appears after Figure 5 in the manuscript. The authors should carefully check the figure order and formatting before publication.

**********

what does this mean?). If published, this will include your full peer review and any attached files.

If you choose “no”, your identity will remain anonymous but your review may still be made public.

**Do you want your identity to be public for this peer review?**  For information about this choice, including consent withdrawal, please see our Privacy Policy

Reviewer #2: No

Reviewer #3: No

**********

[NOTE: If reviewer comments were submitted as an attachment file, they will be attached to this email and accessible via the submission site. Please log into your account, locate the manuscript record, and check for the action link "View Attachments". If this link does not appear, there are no attachment files.]

To ensure your figures meet our technical requirements, please review our figure guidelines: https://journals.plos.org/plosone/s/figures

You may also use PLOS’s free figure tool, NAAS, to help you prepare publication quality figures: https://journals.plos.org/plosone/s/figures#loc-tools-for-figure-preparation.

NAAS will assess whether your figures meet our technical requirements by comparing each figure against our figure specifications.

# Author comment: Author response to Decision Letter 2

**DOI:** 10.1371/journal.pone.0361966.r004

31 Aug 2026

We are grateful for the additional comments by the reviewers. We made every effort to address them as thoroughly as possible in the following.

The Editor suggested the abstract to be revised, reminding us of PLOS ONE’s editorial criteria. We made changes across the manuscript in response to this recommendation (e.g., line:31-36).

Reviewer #2 raised following two concerns that still remain even after the first revision.

1. The left panel of Figure 3c shows that there is substantially no difference between the intensity of soma and detected RNA, indicating that RNA granules were not detected with the MCP-1xGFP probe.

As criteria, ImageJ’s Find Max uses prominence, i.e., intensity above surrounding pixel values (not the noise level as the reviewer suggests), so weak spots comparable to soma background could be detected provided that they are situated within relatively dim regions. However, as the reviewer points out, the spots by MCP-1xGFP are much more ambiguous compared to those by MCP-4xsfGFP. In response to this comment, we now state that the confidence for positive identification as RNA granules is significantly poorer due to lower contrasts (line 137-141).

2. To evaluate whether the GMM analysis yields appropriate results and to perform the analysis correctly, it is necessary to examine the fluorescence intensity distribution of single Actb-MBS molecules, which provide a standard for GMM analysis.

We agree with the reviewer that the uniform discrete spacings between Gaussian peaks do not necessarily prove that they are due to integer mRNA copy numbers and such association would be valid only with a standard of single mRNA molecules. We revised to emphasize this limitation. As a prior art, we include two additional references (Refs. 24 and 25) where the GMM analysis is applied to smFISH images for determining mRNA copy numbers without independent standards, based on an assumption that the cytoplasmic signals represent the intensity of single copy, and how our images are different (line: 158-164).

Reviewer #3 had the following critiques.

1. Therefore, the improved performance cannot yet be clearly attributed to the 4×sfGFP itself.

In response to this comment, we now state that there could be other contributing factors for the improved performance (line: 142-144).

2. The GMM analysis identifies statistical subpopulations but does not directly demonstrate that these populations represent different RNA copy numbers.

This comment is also echoed by Reviewer #2, and we address it as above.

3. The conclusion in Figure 4 that RNA granule movement is “independent of active transport” appears too strong. … no obvious effect was detected under the current experimental conditions.

In response to this suggestion, we removed the phrase from the section title for Figure 4 (line 165) and revised relevant texts (line: 239-240) and added the suggested edit regarding the limitation of the current experimental conditions (line 174-175). It is described in more detail in a paragraph in the Discussion (line 244-249).

4. The authors should carefully check the figure order and formatting before publication.

The formatting of submitted manuscript was done automatically by the PLOS ONE system. We will double check the figure order during resubmission.

Attachment Submitted filename: Response_LimCooper_r2.docx

# Peer review: Decision Letter 2

**DOI:** 10.1371/journal.pone.0361966.r005

**Authors:** Daniel Larson

20 Oct 2026

Amplified MS2 labeling reveals heterogeneous dynamics of RNA granules in the live murine brain ex vivo

PONE-D-26-11425R2

Dear Dr. Lim,

We’re pleased to inform you that your manuscript has been judged scientifically suitable for publication and will be formally accepted for publication once it meets all outstanding technical requirements.

Within one week, you’ll receive an e-mail detailing the required amendments. When these have been addressed, you’ll receive a formal acceptance letter and your manuscript will be scheduled for publication.

An invoice will be generated when your article is formally accepted. Please note, if your institution has a publishing partnership with PLOS and your article meets the relevant criteria, all or part of your publication costs will be covered. Please make sure your user information is up-to-date by logging into Editorial Manager at Editorial Manager® and clicking the ‘Update My Information' link at the top of the page. For questions related to billing, please contact billing support.

If your institution or institutions have a press office, please notify them about your upcoming paper to help maximize its impact. If they’ll be preparing press materials, please inform our press team as soon as possible -- no later than 48 hours after receiving the formal acceptance. Your manuscript will remain under strict press embargo until 2 pm Eastern Time on the date of publication. For more information, please contact onepress@plos.org.

Kind regards,

Daniel R. Larson

Academic Editor

PLOS One

Additional Editor Comments (optional):

I have read the multiple revisions and reviewer comments. In my determination, this article is acceptable for publication. I attach the last round of reviews for the benefit of the authors, and they can make the small textual changes if they choose to do so.

Reviewers' comments:

Reviewer's Responses to Questions

**Comments to the Author**

Reviewer #2: (No Response)

Reviewer #3: (No Response)

**********

2. Is the manuscript technically sound, and do the data support the conclusions?

Reviewer #2: Yes

Reviewer #3: Partly

**********

3. Has the statistical analysis been performed appropriately and rigorously?

Reviewer #2: Yes

Reviewer #3: (No Response)

**********

4. Have the authors made all data underlying the findings in their manuscript fully available?

The PLOS Data policy

Reviewer #2: Yes

Reviewer #3: Yes

**********

5. Is the manuscript presented in an intelligible fashion and written in standard English?

Reviewer #2: Yes

Reviewer #3: Yes

**********

Reviewer #2: The authors have substantially improved the manuscript in response to the previous comments. In particular, the limitations of the MCP-1×GFP detection and of the GMM analysis are now more clearly acknowledged. I have two remaining comments regarding the consistency of these revised descriptions.

1. Regarding the comparison between MCP-1×GFP and NLS-MCP-4×sfGFP in Figure 3, the revised manuscript now appropriately states that, because of the low contrast of the spots visualized by MCP-1×GFP, “the confidence for positive identification as RNA granules was poor, thus likely inaccurate.” However, the manuscript subsequently states that “there were nearly thirteen times more RNA particles with NLS-MCP-4×sfGFP compared to MCP-1×GFP”. These two statements are not fully consistent. If the puncta detected with MCP-1×GFP cannot be reliably identified as RNA granules, the 13-fold difference should not be interpreted as a quantitative difference in the number of RNA particles. I therefore suggest revising this statement to describe the difference in the number of detected puncta or peaks, rather than RNA particles, and clarifying that the 13-fold value does not necessarily represent the true ratio of RNA granule numbers.

2. Regarding the GMM analysis, the revised manuscript now appropriately acknowledges that, without an independent single-mRNA intensity standard, the approximately uniform spacing between Gaussian components cannot be verified to represent integer multiples of Actb mRNA molecules. This revision adequately addresses the major concern raised previously. However, the phrase “quantal intensities” is still used to describe the fitted components. Because this wording may imply a physically defined quantization corresponding to discrete RNA copy numbers, it appears stronger than what is supported by the present data. I suggest replacing “quantal intensities” with a more neutral description, such as “approximately regularly spaced intensity modes” or “multiple intensity subpopulations with approximately equal mode spacing.”

Reviewer #3: Most of my concerns have been addressed. However, one issue should be resolved before acceptance. The authors now acknowledge this confound in the Results (lines 142-144), but the conclusions that depend on it have not been revised accordingly. The Discussion (lines 218-220) still presents this gain as an advance attributable to the probe. And the Fig 3 legend should state explicitly that the two conditions differ in promoter, NLS, serotype and titer.

**********

what does this mean?). If published, this will include your full peer review and any attached files.

If you choose “no”, your identity will remain anonymous but your review may still be made public.

**Do you want your identity to be public for this peer review?**  For information about this choice, including consent withdrawal, please see our Privacy Policy

Reviewer #2: No

Reviewer #3: No

**********
